# Supplementary material for: Causal Relationship between Aging and Anorexia Nervosa: A White-Matter-Microstructure-Mediated Mendelian Randomization Analysis
Source: Biomedicines. 2024 Aug 16;12(8):1874. doi: 10.3390/biomedicines12081874 (PMC11351342; doi:10.3390/biomedicines12081874)
Supplement: Supplementary file 1 [file biomedicines-12-01874-s001.zip › Supplementary Figure/Figure S4/Singe SNP plot of MR analysis for TL to ubm-b-1636.pdf]

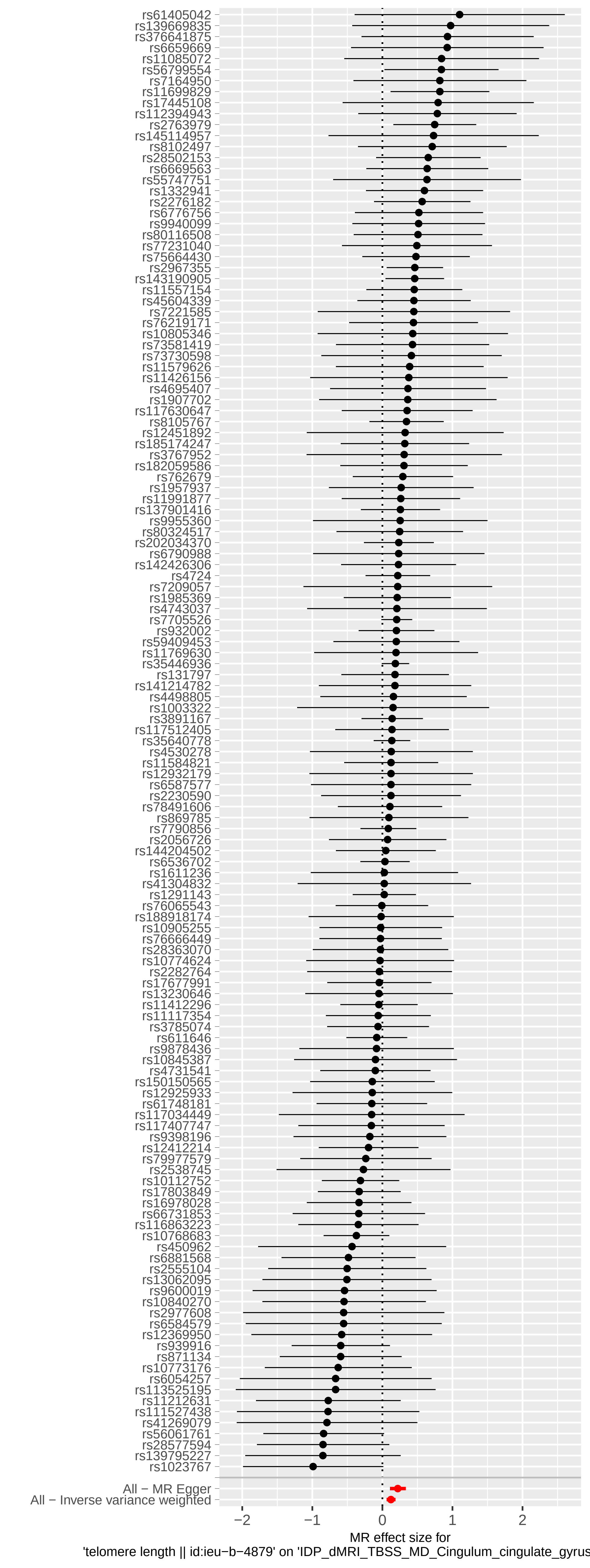
rs61405042
rs139669835
rs376641875
rs6659669
rs11085072
rs56799554
rs7164950
rs11699829
rs17445108
rs112394943
rs2763979
rs145114957
rs8102497
rs28502153
rs6669563
rs55747751
rs1332941
rs2276182
rs6776756
rs9940099
rs80116508
rs77231040
rs75664430
rs2967355
rs143190905
rs11557154
rs45604339
rs7221585
rs76219171
rs10805346
rs73581419
rs73730598
rs11579626
rs11426156
rs4695407
rs1907702
rs117630647
rs8105767
rs12451892
rs185174247
rs3767952
rs182059586
rs762679
rs1957937
rs11991877
rs137901416
rs9955360
rs80324517
rs202034370
rs6790988
rs142426306
rs4724
rs7209057
rs1985369
rs4743037
rs7705526
rs932002
rs59409453
rs11769630
rs35446936
rs131797
rs141214782
rs4498805
rs1003322
rs3891167
rs117512405
rs35640778
rs4530278
rs11584821
rs12932179
rs6587577
rs2230590
rs78491606
rs869785
rs7790856
rs2056726
rs144204502
rs6536702
rs1611236
rs41304832
rs1291143
rs76065543
rs188918174
rs10905255
rs76666449
rs28363070
rs10774624
rs2282764
rs17677991
rs13230646
rs11412296
rs11117354
rs3785074
rs611646
rs9878436
rs10845387
rs4731541
rs150150565
rs12925933
rs61748181
rs117034449
rs117407747
rs9398196
rs12412214
rs79977579
rs2538745
rs10112752
rs17803849
rs16978028
rs66731853
rs116863223
rs10768683
rs450962
rs6881568
rs2555104
rs13062095
rs9600019
rs10840270
rs2977608
rs6584579
rs12369950
rs939916
rs871134
rs10773176
rs6054257
rs113525195
rs11212631
rs111527438
rs41269079
rs56061761
rs28577594
rs139795227
rs1023767
All − MR Egger
All − Inverse variance weighted
−2
−1
0
1
2
MR effect size for
'telomere length || id:ieu−b−4879' on 'IDP_dMRI_TBSS_MD_Cingulum_cingulate_gyrus
